# 8q24 sequence variants in relation to prostate cancer risk among men of African descent: A case-control study

**DOI:** 10.1186/1471-2407-10-334

**Published:** 2010-06-28

**Authors:** Marnita L Benford, Tiva T VanCleave, Nicole A Lavender, Rick A Kittles, LaCreis R Kidd

**Affiliations:** 1 Department of Pharmacology & Toxicology, University of Louisville, Louisville, KY, USA; 2 Cancer Prevention Program, James Graham Brown Cancer Center, University of Louisville, Louisville, KY, USA; 3 University of Illinois, Department of Medicine, Chicago, IL, USA

## Abstract

**Background:**

Human chromosome 8q24 has been implicated in prostate tumorigenesis.

**Methods:**

Consequently, we evaluated seven 8q24 sequence variants relative to prostate cancer (PCA) in a case-control study involving men of African descent. Genetic alterations were detected in germ-line DNA from 195 incident PCA cases and 531 controls using TaqMan polymerase chain reaction (PCR).

**Results:**

Inheritance of the 8q24 rs16901979 T allele corresponded to a 2.5-fold increase in the risk of developing PCA for our test group. These findings were validated using multifactor dimensionality reduction (MDR) and permutation testing (p = 0.038). The remaining 8q24 targets were not significantly related to PCA outcomes.

**Conclusions:**

Although compelling evidence suggests that the 8q24 rs16901979 locus may serve as an effective PCA predictor, our findings require additional evaluation in larger studies.

## Background

Prostate cancer (PCA) accounts for 25% of all diagnosed cancer cases and was the second leading cause of cancer-related deaths in 2009 among U.S. men [1]. Well-established risk factors of PCA include age, family history of PCA, and ethnicity. African American men carry a substantial portion of the disease burden and are 1.6- and 2.4-fold more likely to be diagnosed with PCA and die from disease, respectively, relative to their Caucasian counterparts [2]. This disparity may be partially attributed to a combination of genetic predisposition and environmental exposures. Despite evidence supporting the role of genetics in PCA etiology, the search for common sequence variants as susceptibility markers has been largely unrewarding. However, recent genome-wide association studies (GWAS) indicate that chromosome 8q24 is a region of interest for further exploration of health disparity markers relative to PCA incidence and outcomes.

Several genetic variants or single nucleotide polymorphisms (SNPs) in the 8q24 region have been associated with PCA risk in Caucasians; however, similar studies on African Americans are only represented in a handful of published reports [3-8]. Amundattoir and co-workers (2006) demonstrated that the DG8S737- 8 microsatellite was significantly associated with PCA risk and disease progression in men of European and African descent [5]. The association signal of DG8S737- 8 in Caucasians has been captured by rs1447295 or one of its linkage disequilibrium equivalents. This was not the case for African Americans, which suggests that the DG8S737- 8 microsatellite was either associated with increased PCA risk or is significantly correlated with other 8q24 sequence variants not detected in a GWAS scan or HapMap project. Two subsequent studies provide compelling evidence that two 8q24 sequence variants, rs6983561 and rs16901979, may be important PCA indicators for Caucasians and African Americans [3,6]. Although the risk estimates for rs16901979 were similar between Caucasians and African Americans, this marker's higher prevalence among African Americans may partially explain the greater PCA incidence in this high-risk group. In fact, Gudmundson and co-workers (2007) suggested that the rs16901979 SNP alone could account for a large fraction of PCA risk among African Americans, as they had a considerably high population-attributable risk associated with this marker (24%) relative to Caucasians [7]. Two other sequence variants, rs4242382 and rs4242384, were significantly related to organ-confined or aggressive disease when compared to disease-free men of European descent [9-11].

Unfortunately, the impact of these two loci and other 8q24 markers (e.g., rs6983561, rs1447295, rs11934905, rs16901979, and rs10090154) on PCA risk among men of African descent remains largely unknown. Although the 8q24 rs10090154T allele confers a 1.7-fold increase in PCA risk in a small case-control study set involving African Americans (85 cases and 149 disease-free men), this locus only reached borderline significance (OR = 1.69; 0.92-3.11).

To clarify the role of 8q24 sequence variants in PCA susceptibility among men of African descent, we sought to confirm previous reports and generate new data on the role of seven 8q24 sequence variants in PCA risk among 864 men of African descent (195 cases and 531 disease-free men). To overcome sample size issues and control for multiple comparisons, we used a multifactor dimensionality reduction (MDR) algorithm along with permutation testing. This approach will aid future studies that analyze different sequence variants within the 8q24 region to decode health disparities in high-risk subgroups, especially PCA risk in men of African descent.

## Methods

### Study population

Between 2001 and 2005, 864 unrelated male residents were recruited from the Washington, D.C. and Columbia, SC areas through the Howard University Hospital (HUH) Division of Urology or PCA screening programs. The study population of men of African descent (i.e., self-reported African Americans, East African Americans, West African Americans, and Afro-Caribbean Americans) consisted of 195 incident PCA cases and 531 controls. PCA patients between the ages of 41 and 91 were diagnosed within one year of enrollment. Following a visit to the HUH Division of Urology for an annual PCA screening exam or urinary symptoms, incident PCA cases were identified by a urologist using a transrectal ultrasound-guided biopsy [12]. Biopsy cores were reviewed by members of the Department of Pathology at the Howard University College of Medicine. PCA cases were classified according to a well-established Gleason scoring system [13]. Inclusion criteria of controls included men older than 45 with a low prostate specific antigen (PSA) level ≤ 4.0 ng/ml and normal digital rectal exams (DREs) or biopsies. Individuals were excluded from the current study if: they failed at least one diagnostic test (i.e., PSA > 4.0 and/or irregular PSA), even though they had a normal biopsy; or were diagnosed with benign prostatic hyperplasia (BPH). Clinical characteristics including Gleason score, PSA and age at diagnosis/enrollment were obtained from medical records, as summarized in Table 1. The median age of 65.0 for PCA cases (range: 41-91) was significantly older than that of disease-free men [median = 53 yrs (45-89)]. Tumor grade, ranging from 4-10, was collected for 59% of the cases (n = 115). All study participants had DNA extracted from whole blood and provided written informed consent for participation in genetic analysis studies under a protocol approved by Howard University, the HUH Division of Urology, and the University of Louisville Institutional Review Board.

**Table 1:** Patient and Tumor Characteristics

Characteristics	Cases	Controls	**P-value**^**a**^
**No. of Participants, n**	195	531	---

**Age (yrs)**			
Median (range)	65.0 (41-91)	53.0 (45-89)	< 0.0001

**PSA in ng/ml**			
Median (range)	7.0 (0.01-5,000)	1.08 (0.01-3.9)	< 0.0001
< 2.0, n (%)	43 (22.0)	464 (87.4)	
2.0-4.0	12 (6.2)	67 (12.6)	
> 4.0	140 (71.8)	0 (0.0)	

**Gleason Score **n (%)			
4	15 (13.0)		
5	11 (9.6)		
6	31 (26.9)		
7	37 (32.2)		
8	5 (4.4)		
9	12 (10.4)		
10	4 (3.5)		

**Global West African Ancestry**			
**Median (SD)**	0.788 (0.253-0.937)	0.734 (0.255-0.946)	0.0273

Abbreviations: PSA, prostate specific antigen

^a^Differences in median age, PSA levels (ng/ml), and West African Ancestry levels comparing cases and controls were tested using the Wilcoxon rank sum.

### TaqMan allelic discrimination of 8q24 sequence variants

Polymorphisms detected in the 8q24 region were ascertained using TaqMan Polymerase Chain Reaction (PCR) allelic discrimination assays. The following seven alleles were detected: **(1) **rs6983561 (A > C); **(2) **rs1447295 (G > T); **(3) **rs4242384 (A > C); **(4) **rs4242382 (G > A); **(5) **rs11934905 (G > A); **(6) **rs16901979 (G > T); and **(7) **rs10090154 (G > A). Each allelic discrimination assay contained approximately 40 ng of germ-line DNA, 1× Universal Master Mix (Applied Biosystems, Foster City, CA), a 40× mixture containing 900 nM of each primer (forward and reverse), and 200 nM of each probe (FAM and VIC) to comprise a 5 μl reaction. To facilitate amplification of regions containing the aforementioned 8q24 SNPs, primers and probes were designed in our laboratory using sequences provided by NCBI [14] and placed into the FileBuilder software (Applied Biosystems). PCR reactions were carried out using the ABI Prism 7900HT Sequence Detection System (Applied Biosystems). The thermocycling settings consisted of two holds at 50°C for 2 min and 95°C for 10 min, followed by 40 cycles at 95°C for 15 sec/cycle and 1 min at 60°C. The fluorescent intensity emitted from the probes was measured using the ABI 7900 sequence detection system and assigned genotypes with the SDS 2.2.1 software (Applied Biosystems). To minimize misclassification bias, laboratory technicians were blinded to the case status of study participants. Based on 24 non-DNA template controls per batch analysis, the percent cross-contamination during sample handling was less than 4.7%. Duplicate genotyping was performed on 72 randomly selected samples for quality control purposes, resulting in concordance rates of ≥ 97.5%. The genotype call rates ranged between 89.6 and 96.2% across the seven SNPs with a median value of 92.3%. Subjects (n = 62) with 4 or more missing SNP values across the 7 SNPs were removed from the final analysis.

### Ancestry Markers

One hundred ancestry autosomal markers were included to account for potential population stratification among our admixed population of self-reported African-Americans, West African Americans, East African Americans, and Afro-Caribbean Americans, as previously described [15]. Study participants were grouped from lowest to highest genetic West African ancestry, with scores ranging from 0 to 100%. This marker was assembled using DNA from self-identified African-Americans (Coriell Institute for Medical Research, n = 96), Yoruban West Africans (HapMap, n = 60), West Africans (Bantu and Nilo Saharan speakers, n = 72), Europeans (New York City, n = 24), and Centre d'Etude du Polymorphisme Humain (CEPH) Europeans (HapMap Panel, n = 60). Individuals with a high degree of West African ancestry greater than or equal to 25% were included in the current study.

### Screening for single gene markers predictive of PCA risk using conventional Logistic Regression (LR) Analysis

To assess whether inheritance of at least one variant 8q24 allele was associated with an elevated risk of developing PCA, we tested for significant differences in the distribution of seven 8q24 genotypes between 195 cases and 531 controls using the chi-square test of homogeneity. Associations between PCA risk and 8q24 sequence variants, expressed as odds ratios (ORs) and corresponding 95% confidence intervals (CIs), were estimated using unconditional multivariate LR models adjusted for potential confounders (age, PSA, and West African ancestry). All reported risk estimates and 95% CIs for the selected 8q24 loci used the following as reference genotypes: **(1) **rs6983561 (A/A); **(2) **rs1447295 (G/G); **(3) **rs4242384 (A/A); **(4) **rs4242382 (G/G); **(5) **rs11934905 (G/G); **(6) **rs16901979 (G/G); and **(7) **rs10090154 (G/G). For rs11934905, hetero- and homozygotes (AA + GA) were combined due to the low frequency of the minor allele. Test for trend included genotypes as ordinal variables. Statistical significance was assessed using a P-value < 0.05. All chi-square tests and LR analyses were conducted using the SAS 9.1.3 software (SAS Institute Inc., Cary, NC).

### Validation of main effects using MDR

Multifactor dimensionality reduction (MDR) was used to evaluate and validate main effects associated with PCA risk. This algorithmic tool aids in the identification of high-risk markers using a cross validation strategy to estimate the classification and prediction accuracy of individual factor models [16,17]. MDR is a data-mining platform that readily overcomes sample size limitations often encountered by parametric statistical methods (e.g., LR analysis) by collapsing high-dimensional genetic data into a single dimension. For this study, single factor loci were classified into high-risk and low-risk groups, which permitted the investigation of individual effects of variant 8q24 alleles in relation to PCA risk using a cross-validation and permutation testing scheme. MDR was utilized to generate a single model that maximized the number of individuals with the proper risk assignment. The best 8q24 sequence variant was selected among the seven loci that minimized the prediction error as well as maximized the cross validation consistency (CVC) and average testing accuracy (ATA). To evaluate the number of times the same single factor model was identified in each possible 9/10^ths ^of the data, the average CVC (based on a scale from 0-100%) from the observed data was compared to the distribution of average consistencies under the null hypothesis of no association. Validation of models as effective predictors of PCA susceptibility was derived empirically from 10,000 permutations. This approach accounted for multiple testing issues as long as the entire model fitting procedure was repeated for each randomized dataset to provide an opportunity to identify false-positives. We considered MDR permutation results to be statistically significant at the 0.05 level.

## Results

### Patient & Tumor Characteristics

The patient and tumor characteristics in the current study are summarized in Table 1. Cases were significantly older than controls and had higher PSA levels. There was significant difference in median West African genetic ancestry estimates when comparing cases and controls (P = 0.0273).

### Prevalence of 8q24 variant alleles among men of African descent

Our laboratory successfully completed the analysis of seven 8q24 sequence variants among 864 men of African descent (195 PCA cases and 531 controls). Inheritance of at least one minor 8q24 minor allele was fairly common among controls with frequencies ranging between 4.0 and 66.3%, as detailed in Table 2. These findings were comparable to those observed in other men of African descent sub-groups [3,18-20,14].

**Table 2:** Association between 8q24 Sequence Variants and Prostate Cancer Risk.

8q24	Cases	Controls	**OR**_**crude**_	**OR**_**adj**_	**P-Value**_**crude**_^**‡‡**^	P-Trend
	n (%)	n (%)	**(95% CI)**^**†**^	**(95% CI) **^**††**^		
rs6983561						
AA	48 (25.8)	171 (33.6)	1.00	1.00	0.0783	0.0246
AC	88 (47.3)	232 (45.7)	1.35 (0.90 - 2.02)	1.78 (1.10 - 2.89)	0.1436	
CC	50 (26.9)	105 (20.7)	1.70 (1.07 - 2.70)	1.99 (1.15 - 3.46)	0.0258	
AC + CC	138 (74.2)	337 (66.3)	1.46 (1.00 - 2.13)	1. 85 (1.18 - 2.91)	0.0486	

rs1447295						
GG	86 (45.5)	237 (45.3)	1.00	1.00	0.8742	0.8451
GT	77 (40.7)	221 (42.3)	0.96 (0.67 - 1.37)	0.82 (0.55 - 1.26)	0.8239	
TT	26 (13.8)	65 (12.4)	1.10 (0.66 - 1.85)	0.92 (0.49 - 1.70)	0.7121	
GT + TT	103 (54.5)	286 (54.7)	0.99 (0.71 - 1.39)	0.86 (0.45 - 1.61)	0.9647	

rs4242384						
AA	122 (63.2)	383 (73.1)	1.00	1.00	0.0135	0.0515
AC	65 (33.7)	120 (22.9)	1.70 (1.18 - 2.45)	1.50 (0.99 - 2.28)	0.0043	
CC	6 (3.1)	21 (4.0)	0.90 (0.35 - 2.27)	0.91 (0.32 - 2.53)	0.8188	
AC + CC	71 (36.8)	141 (26.9)	1.58 (1.11 - 2.25)	1.41 (0.95 - 2.11)	0.0101	

rs4242382						
GG	85 (40.0)	244 (48.1)	1.00	1.00	0.6002	
GA	80 (41.5)	191 (37.7)	1.20 (0.84 - 1.72)	1.12 (0.74 - 1.69)	0.3148	0.4655
AA	28 (14.5)	72 (14.2)	1.12 (0.68 - 1.84)	1.05 (0.60 - 1.86)	0.6671	
GA + AA	108 (56.0)	326 (51.9)	1.18 (0.85 - 1.65)	1.01 (0.75 - 1.62)	0.3332	

rs11934905						
GG	173 (94.5)	500 (96.0)	1.00	1.00	0.4161	---
GA + AA	10 (5.5)	21 (4.0)	1.38 (0.64 - 2.98)	1.83 (0.54 - 6.24)		

rs16901979						
GG	45 (23.4)	188 (36.7)	1.00	1.00	0.001	0.0002
GT	97 (50.5)	237 (46.3)	1.71 (1.14 - 2.56)	2.28 (1.42 - 3.69)	0.0089	
TT	50 (26.1)	87 (17.0)	2.40 (1.49 - 3.87)	3.02 (1.72 - 5.31)	0.0003	
GT + TT	147 (76.6)	324 (63.3)	1.90 (1.30 - 2.77)	2.49 (1.58 - 3.92)	0.0001	

rs10090154						
GG	124 (65.6)	357 (70.7)	1.00	1.00	0.2878	0.2878
GA	59 (31.2)	131 (25.9)	1.30 (0.90 - 1.88)	1.11 (0.72 - 1.69)	0.1676	
AA	6 (3.2)	17 (3.4)	1.02 (0.39 - 2.64)	0.88 (0.30 - 2.57)	0.9737	
GA + AA	65 (34.4)	148 (29.3)	1.26 (0.89 - 1.81)	1.08 (0.72 - 1.63)	0.1914	

^†^Associations were determined using multivariate LR models to estimate the risk of developing PCA.

^††^Risk estimates adjusted for age (continuous variable) and West African Ancestry (continuous variable).

^‡‡^Differences in the frequency of variant and referent genotypes between cases and controls were determined using the chi-square test of association and a significance level of 0.05.

### Association between 8q24 and PCA

The independent effects of genetic variations detected within the 8q24 region were analyzed relative to PCA susceptibility using MDR and unconditional LR multivariate models adjusted for age and West African ancestry (Table 2). We evaluated whether inheritance of at least one minor 8q24 allele, e.g., rs6983561 (AC + CC), rs1447295 (GT + TT), rs4242384 (AC + CC), rs4242382 (GA + AA), rs11934905 (GA + AA), rs16901979 (GT + TT), or rs10090154 (GA + AA), was associated with PCA risk. We observed significant main effects of sequence variants localized to chromosome 8q24 in relation to PCA risk. Notably, individuals who possessed the rs6983561 C (OR = 1.85; 95% CI = 1.18-2.91; p = 0.0486; p for trend = 0.0246) or the rs16901979 T (OR = 2.5; 95%CI = 1.58-3.92; p = 0.0001; p for trend = 0.0001) alleles had a 1.85- to 2.5-fold increase in the risk of developing PCA. Interestingly, statistical significance was preserved for the rs16901979T allele after adjusting for potential confounders (e.g., age and West African Ancestry) and multiple comparisons. In fact, MDR modeling complemented with permutation testing identified 8q24 rs16901979 as the best single factor predictor of PCA risk (p = 0.038), as noted in Table 3.

**Table 3:** Multifactor Dimensionality Reduction Models for 8q24 rs16901979 Sequence Variants

Best Model	Cross Validation Consistency	Average Testing Accuracy	Permutation Testing p-value
rs16901979	10/10	0.557	0.038

Multifactor dimensionality reduction (MDR) model is considered statistically significant (p < 0.05) if the average testing accuracy is greater than testing accuracy cut-off based on 10,000-fold permutations of all possible single factor loci.

## Discussion

Sequence variants localized to the 8q24 region have recently been associated with numerous cancer sites [4,18,21,22]. In the current study, we evaluated the relationship between seven sequence variants and the risk of developing PCA and aggressive disease among 195 cases and 531 disease-free study participants. Our analyses revealed a statistically significant 2.5-fold increase in the risk of developing PCA among men of African descent who carry the rs16901979 T allele. This relationship remained important after adjusting for potential confounders and multiple comparisons. None of the other six sequence variants we analyzed were significantly related to PCA risk.

Our findings on the link between rs16901979 and PCA risk were validated using MDR, which remains effective with relatively small sample sizes. In addition, this observed correlation is further strengthened by two out of three other studies involving African American men. These two independent studies both revealed a 1.3- to 1.5-fold increase in the risk of developing PCA with 95% CIs that exclude unity [6,7].

Several of the evaluated 8q24 SNPs may be more appropriate as PCA predictors among men of European- rather than African descent [3,5,6,11,23]. The rs4242384, rs4242382, rs1447295, and rs10090154 sequence variants were not significantly associated with PCA outcomes among men of African descent in the current study and/or published reports, but these markers appear to play a role in PCA risk or aggressive disease among men of European descent [3,5-7,9,11,19,23-25]. Zheng and co-workers (2007) observed a 1.3- to 1.9-fold increase in PCA risk among European carriers of one or two rs4242382 A alleles (OR = 2.90; 95% CIs = 1.02 - 8.25 and OR = 2.90; 95% CIs = 1.02 - 8.25, respectively) [10,11]. Moreover, Cussenot and co-workers (2008) also demonstrated a strong relationship between organ-confined (T_1_-T_2_, N_0 _M_0_) and advanced (T_3_-T_4_, N_1_M_1_) PCA among carriers of 8q24 rs4242382A and rs4242384C alleles [9]. To our knowledge, there are no published reports on the relationship between the rs11934905 SNP localized to the 8q24 chromosome in relation to PCA among men of European or African descent.

We have considered the strengths and limitations of the current study. MDR controls for multiple comparisons and spurious risk estimates by using a cross validation and permutation testing scheme as a built-in feature. Misclassification by case status is also a potential limitation for this study. There is a possibility that some men who presented as disease-free following the initial diagnostic evaluation may eventually develop PCA. In an attempt to ease case-status misclassification, controls with at least one abnormal diagnostic test (i.e., PSA > 2.0 ng/ml or irregular DRE) underwent multiple core needle biopsies. Those with an abnormal biopsy were reclassified as cases. Men who received a normal biopsy test but had an abnormal PSA (> 4.0 ng/ml) and/or irregular DRE (n = 48) were excluded because we could not predict with any level of certainty whether they would eventually develop PCA. For similar reasons, we also excluded individuals (n = 65) who were diagnosed with BPH following biopsy. Notably, after close inspection of PCA tissue, it is feasible to overlook a microscopic nodule that can later develop into cancer [26]; however, this is an issue that plaques many cancer epidemiology studies. If controls in our study population were still misclassified after undergoing a PSA test, DRE, and/or multiple core needle biopsies, then we may expect our calculated risk estimates to slightly underestimate the relationship between the selected 8q24 loci and PCA susceptibility.

Another challenge for genetic epidemiology studies involving study participants of African descent is their unique population history of gene flow from divergent populations [27,28]. The current study adjusted single locus models for population admixture using a West African ancestry score assigned to each study participant. Utilization of ancestry markers helps to eliminate misclassification of study participants based on confusion related to self-identified race/ethnicity (SIRE). Our findings suggest that adjusting our risk estimates for West African ancestry did not significantly change the risk estimates relative to unadjusted models; if anything, it makes them more precise to the nearest tenth.

Finally, it is feasible that the observed association is related to linkage disequilibrium of rs16901979 with unknown targets in this region. To address this issue, future studies will use next generation sequencing techniques to better consider the complete heterogeneity within this locus relative to PCA outcomes. Emphasis will be placed on sequence variants within region 3, as it is speculated that sequence variants may be related to a 2-fold increase in PCA risk [12,13,29]. Our findings demonstrating a significant relationship between rs16901979 and PCA risk have been submitted for further validation within pooled genetic analyses.

## Conclusions

In summary, the 8q24 rs16901979 locus has a strong genetic linkage to PCA among men of African descent in the current investigation as well as other studies. Future multicenter collaborative efforts will facilitate the identification and validation of a reliable panel of genetic susceptibility biomarkers with the capacity to improve PCA detection and prognosis strategies, ultimately reducing PCA health disparities among all men.

## Competing interests

The authors declare that they have no competing interests.

## Authors' contributions

Taqman PCR reactions were carried out in an ABI Prism 7900HT Sequence Detection System (Applied Biosystems) by MLB, TTV, and NAL. TTV designed the primer and probe sequences for Taqman allelic discrimination assays. MLB and LRK performed statistical analysis and produced drafts of this manuscript. All authors made contributions toward editing the manuscript.

## Pre-publication history

The pre-publication history for this paper can be accessed here:

http://www.biomedcentral.com/1471-2407/10/334/prepub
